# A microcomputer-based automated curve tracer for accurate digitization of paper-recorded spectra

**DOI:** 10.1155/S1463924684000420

**Published:** 1984

**Authors:** J. C. W. G. Bink, H. A. van't Klooster

**Affiliations:** State University of Utrecht Laboratory of Analytical Chemistry Croesestraat 77a Utrecht 3522 AD The Netherlands


Journal of Automaic Chemistry, Volume 6, Number 4 (October-December 1984), pages 210-213

### A microcomputer-based automated curve tracer for accurate digitization of paper-recorded spectra

**J. C. W. G. Bink and H. A. van 't Klooster***

*State University of Utrecht, Laboratory of Analytical Chemistry, Croesestraat 77a, 3522 AD Utrecht, The Netherlands*

### Introduction

Although direct connection of analytical instruments to computers allows on-line acquisition of computer-readable data, there are still many laboratory situations which require off-line digitization of compiled analogue data as a separate step in the process of automatic data analysis. Such a situation applies to existing laboratory compilations of spectra recorded on paper documents, which, particularly as references, may be of great value to the laboratory. Commercially available digitized spectra files can be expensive, often they do not have the desired accuracy, and frequently they represent compounds which are not of interest to the user. On-line recording of new reference spectra may be impossible or impractical: the necessary reference compounds being either unavailable or expensive. So in these situations digitizing the available paper-recorded spectra is an attractive option.

A general discussion on the problems involved in compiling computer-readable spectra libraries is given by Büchi *et al.* [1]. These authors used a writing tablet linked to a PDP minicomputer for semi-automatic digitization of analogue spectra. De Haseth *et al.* [2] designed a computer-interfaced vidicon camera for direct optical encoding of spectra and chemical structure models. Delaney and Uden [3] developed a digitizer, using an X-Y servorecorder and a PDP computer, to encode 500 vapour-phase infra-red spectra. This idea was then adopted by Razinger *et al.* [4] for the digitization of infrared spectra of polymers.

Video systems, however, generally require a high investment. Semi-automatic digitizer systems are less expensive, but are only effective if manual measurement of separated data-points is practicable. It is difficult to maintain a high accuracy when continuously digitizing spectra in small intervals, resulting in a stream of data points. Also, operating a digitizer in data-stream mode is complicated.

In view of the fact that microcomputers and plotters are now available in many laboratories, an automatic curve tracer has been developed using these machines; there are few additional costs. The general idea is to use a plotter as a reading instead of a plotting device, by replacing the pen by an optical reflective sensor, and writing appropriate software to control the sensor and to process the digitized data. This curve tracer finds and follows a curve, while fully digitizing it, with an accuracy which mainly depends on the quality of the plotter. Manual work is limited to marking a spectrum, which enables the curve tracer to determine a starting point and a scale factor.

*Correspondence to Dr van 't Klooster.

### Hardware

A schematic representation of the curve tracer is shown in figure 1. The electronics needed for the optical reflective sensor are shown in figure 2.

The sensor (an HP HEDS-1000 was used) is mounted as a regular pen on a Calcomp 81 plotter. The plotter is interfaced with an Apple II microcomputer using an RS-232 interface at 2400 baud. The necessary power for the sensor is supplied by the Apple, which needs two additional input bits and one output bit. Hence, the game-I/O of the Apple is sufficient. One input bit detects the current status (black/white) of the surface under the sensor; the second input bit is used to determine whether a black line is detected or has been passed since the last start position on the trajectory of the sensor. This important memory function can be reset with the output bit, which is used for generating a negative strobe signal. The serial interface is used to send commands to the plotter to move the sensor to a new position on the plotter bed. Additional interfacing electronics consist of an operational amplifier to transform the signals to TTL level, a Schmitt trigger to transform the signals to block-shaped pulses and some logic to hold information about the temporary status of the scanned surface. The optical reflective sensor, type HP HEDS-1000, consists of a 0·18 mm diameter 700 nm visible LED emitter and a matched I.C. photodetector. The active areas of the emitter and the detector are imaged to a single spot, 4·27 mm in front of the sensor. The reflected signal is sensed directly from the photodiode (Hewlett-Packard documents [5]). Background-lines of colours other than black do not interfere with the detection of black lines. An adjustable resistance is included, enabling the user to control the sensor's sensitivity. The accuracy of the curve tracer is determined by the diameter of the emitted beam of the sensor and by the repeatability and accuracy of the plotter. An average accuracy of 0·2 mm is possible when using a plotter like the Calcomp 81. The speed of the system is determined partly by the hardware configuration, but mainly by the effeciency of the program that controls the curve tracer and by the required accuracy of digitization.

### Software

The software was developed with COMPAS PASCAL, a Pascal compiler designed to run on Z-80-based microcomputers with CP/M operating system, which allows structured programming and combines high-speed operation with convenient direct interfacing capabilities (Poly-Data documents [6]).

The program, 'CURVETRACE', operates as follows. After finding the markers on the spectral document, the width of the

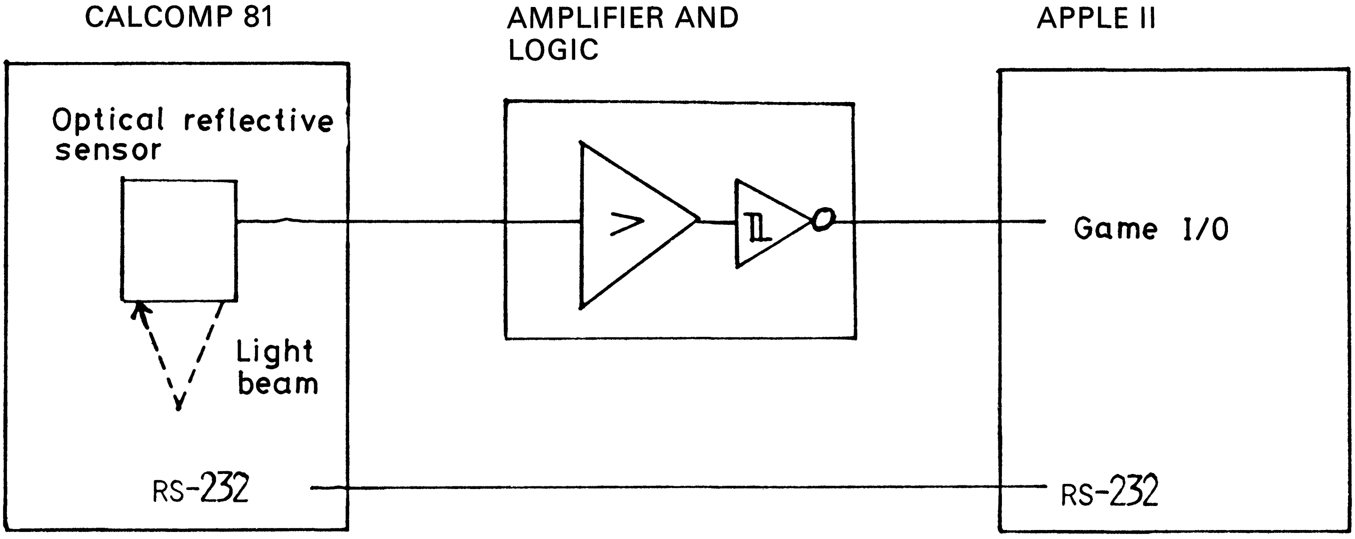

*Figure 1. Schematic representation of the main hardware components of the curve tracer.*

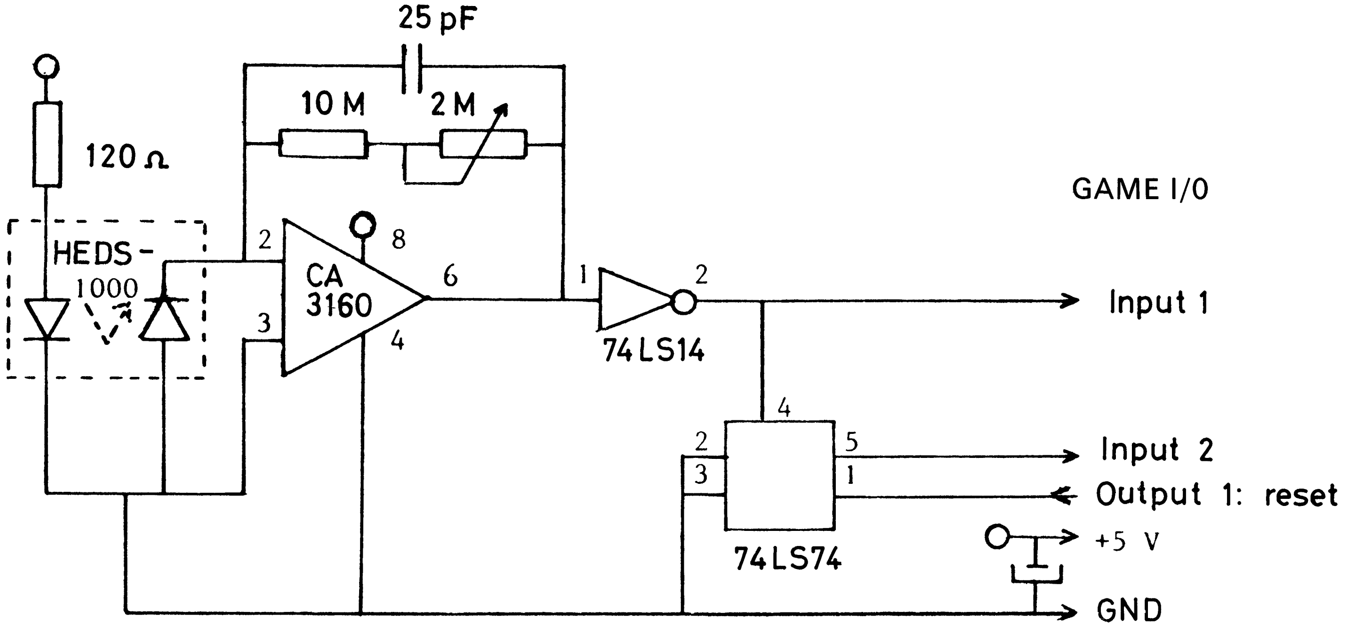

*Figure 2. Electronics required for the optical reflective sensor.*

curve is estimated, and the system starts digitizing at the beginning of the spectral curve. After each step the output signals of the sensor are interpreted and the program determines the next action until the end position is reached. Adequate marks on the paper document enable the program to determine start and end positions and to calculate scale factors. This is often necessary since paper documents tend to be of variable length. Also, it is possible to correct for deviations caused by positioning of documents not completely aligned to the plotter bed; usually these rotations are negligible.

Since the system can generally measure more accurately than allowed by the line-width of a spectral curve, CURVETRACE scans the curve's outer border. Knowing the width of the line means that it is possible to retrospectively calculate the position of the middle of the spectral curve.

The memory signal from the optical sensor is important for special software applications. It enables the curve tracer to scan a trajectory in one step in order to determine whether a black line has been passed. This can lead to fast binary search routines. In combination with the other (temporary) signal the software can determine the position of the sensor with regard to the spectral curve. This deduction, however, poses some problems. For instance, moving the sensor from white to black to white in the direction of the wavelength axis of the spectrum does not give unique information about the position of the sensor. The sensor may have passed the spectral curve, or it may have passed a small peak or spike staying at the same side of the curve as at the starting position. It seems impractical to develop an algorithm which theoretically works 100% error-free, since there still remains a small, but distinct, chance of running into unpredictable traps and hardware failures. Therefore, three error-trap routines have been developed. First, when there is doubt, a quick scan can determine the position of the sensor with regard to the spectral curve. Secondly, when moving the sensor into a certain direction while searching the spectral curve, the search distance will be limited; if this limit is exceeded then the program must be in error, in which case the scan direction is inverted to resume the process of digitization. Thirdly, the program can be stopped and resumed (even from the start) at any point in order to correct for errors or irregularities, such as missing marks, spots or lines on the document, or interrupts in the spectral curve. After processing a complete spectrum the data are stored on a diskette.

Replotting stored results is possible by running a plot program after replacing the sensor with a pen. A quick check on the quality of the results is then provided by visual comparison of original and reconstructed (replotted) spectra. Further processing of the stored data may be carried out on the micro, or on a laboratory computer.

### Results and discussion

The curve tracer was tested on 150 infra-red spectra of purified liquid hydrocarbons, recorded on a double-beam spectrometer with an accuracy of the frequency of 5 cm$^{-1}$ in the range 4000–2000 cm$^{-1}$, 2 cm$^{-1}$ in the range 2000–600 cm$^{-1}$, and of 2% of the transmittance.

The spectra were marked and digitized from 4000 cm$^{-1}$ to 600 cm$^{-1}$. Since these infra-red spectra are approximately $15 \times 48$ cm and the plotter bed is of A3 format, the spectra were digitized in two parts. Marking of the spectra was done by drawing lines of 0·5 mm width and 1 cm length at the following points:

(1) Vertically at 0%T and 4000 cm$^{-1}$, at 0%T and 2000 cm$^{-1}$ and at 0%T and 600 cm$^{-1}$.

(2) Horizontally at 0%T and 4000 cm$^{-1}$, at 0%T and 2000 cm$^{-1}$, at 102%T and 4000 cm$^{-1}$ and at 102%T and 2000 cm$^{-1}$.

The curves were digitized with intervals of 0·2 mm along the wavelength axis, if the transmittance value was between 0 and 90%T. (To save time the intervals can be expanded to 0·5 mm if the transmittance value is greater than 90%T.) After each step along the wavelength axis the corresponding transmittance value was first roughly found and then determined with increments of 0·1 mm. This can result in a maximum of 2400 points per spectrum, depending on the position of the base-line of the spectrum. In this way a spectrum can be digitized, on average, in 15 to 20 min.

Replotting the digitized spectra has resulted in pictures that look like nearly exact copies of the original spectra. A typical example is shown in figure 3.

Only very sharp fluctuations which fall within the mazes of the measurement intervals are slightly topped. Also, without a correction for the width of the original curves, the digitized versions tend to have slightly broader profiles, which was to be expected as the outer sides of the curves were digitized.

The repeatability of the plotter used was 0·1 mm, which results in an accuracy of 1 cm$^{-1}$ in the range of 4000–2000 cm$^{-1}$ and 0·5 cm$^{-1}$ in the range of 2000–600 cm$^{-1}$. For transmittance values this would result in an error less than 0·02%T.

Additional errors, however, will be made since the transmittance values are measured. The magnitude of these errors will increase with the steepness of the curve and is caused by the finite size of the lightspot of the sensor. When measuring perpendicularly on a black line the deviation will usually not exceed 0·2 mm.

As for the speed of the system: the computer will interpret the sensor's signals after a fluctuating time interval, when the sensor has reached its programmed position. When a hardware signal would be available, an optimal (critical) timing would be possible. We used a less optimal software timing. Further improvements in speed can be obtained by using a higher baud-rate or a parallel interface and obviously by specifying wider frequency intervals.

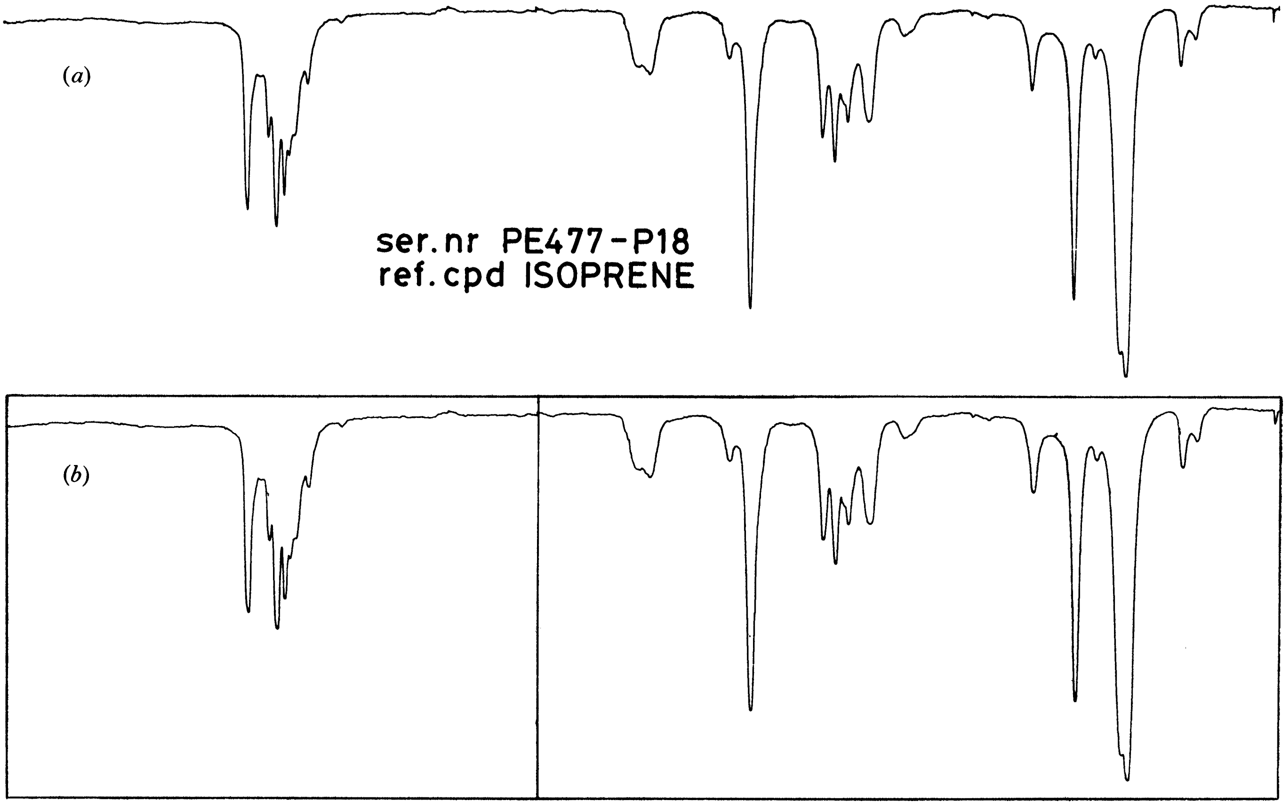

*Figure 3 (a). Original infra-red spectrum of isoprene, recorded on paper. (b). Replotted spectrum from digitized spectrum (a) as produced by the curve tracer. Digitization was carried out in two parts (separated by the vertical line), because the original spectrum was too long for the plotter bed.*

In conclusion, it has been shown that is is possible to build a low-cost, fully automated curve-tracing digitizer, based on a microcomputer, a plotter and a high-resolution optical reflective sensor. The digitizing accuracy of the system mainly depends on the quality of the plotter, and on the resolution of the optical reflective sensor. Apart from the efficiency of the software, the speed of the system is principally controlled by the required accuracy.

### Acknowledgements

The authors wish to thank Mr H. Erkelens and Mr C. van der Lee for valuable technical assistance.

### References

1. Büchi, R., Clerc, J. T., Jost, Ch., Koenitzer, H. and Wegmann, D., *Analytica Chimica Acta*, **103** (1978), 21.

2. De Haseth, J. A., Woodward, W. S., and Isenhour, T. L., *Analytical Chemistry*, **48** (1976), 1513.

3. Delaney, M. F., and Uden, P. C., *Analytical Chemistry*, **50** (1978), 2156.

4. Razinger, M., Penca, M. and Zupan, J., *Analytical Chemistry*, **53** (1981), 107.

5. Hewlett-Packard, *Optoelectronics Designer's Catalog* (1983), 184–189 and 624–643.

6. Poly-Data Microcenter ApS, *Operating Manual—Common Pascal Language System* (Copenhagen, 1983).

### PITTSBURGH

### *Computing*

On 25 February 1985, at the annual Pittsburgh Conference and Exposition, a tutorial workshop is to be presented on PCs in the laboratory, this will be followed by a session on the elements of the new instrumentation science, from microcomputer subsystems in instruments to expert systems for acquisition and interpretation of data. On 26 February the Society for Analytical Chemists of Pittsburgh Award Symposium, 'Chemometrics and process analytical chemistry' honouring Professor Bruce Kowalski, will be held. This will cover the impact of computing in industrial processes and the new opportunities for industrial analytical chemists. Additionally, a panel discussion, held as part of the Monday afternoon symposium, will give the audience the opportunity to discuss developments in their own laboratories.

*More information from David R. Weill III, Shady Side Academy, 423 Fox Chapel Road, Pittsburgh, Pennsylvania 15238, USA.*

### *Exhibition*

The 1985 Pittsburgh Conference Exposition will again present an exhibit of modern laboratory equipment and instrumentation for analytical chemistry and applied spectroscopy. In 1984, 610 American and foreign companies exhibited their products in 1607 booths and 34 seminar rooms. The Exposition will be open from Monday morning, 25 February to Thursday afternoon, 28 February 1985.

*For information regarding the exposition or reservation of space, please contact: Peter M. Castle, Exposition Chairman, Pittsburgh Conference, 437 Donald Road, Dept. WT, Pittsburgh, Pennsylvania 15235, USA.*

### 'FOOD TOXICOLOGY'

A new book from Taylor & Francis; it is edited by G. Gibson and R. Walker and copies (price *c.* £40·00) will be available in Spring 1985. Articles in this multi-author volume include:

### *Food safety*

Allocation of priorities—where do the real risks lie?
New approaches to toxicity testing
Evaluation of major food ingredients
Allergies and idiosyncratic responses
Potential 'loopholes' in the routine safety evaluation of infant feeding formulae: possible lessons from the preparation of sow's milk substitutes for piglets.

### *Legislation*

Control of food additives and contaminants in the United Kingdom
Control of food additives and contaminants, the EEC situation
Control of food additives and contaminants, the Canadian and United States position
The form and role of food surveillance
The role of epidemiology in identifying dietary hazards.

### *Problems*

Artificial sweetners: the long running saga
Antioxidants: carcinogenisity and modifying activity in tumorigenesis
The problem of food additives: food colours
Toxicology of caramel colours: current status
Preservatives
Flavourings—a question of priorities
Hyperactivity and food additives: facts or myths.

### *Contaminants*

Evaluation of risks from pesticide residues in food
Heavy metal residues
Contamination from packaging materials
Growth promoters: residues in food
Spain: toxic oil syndrome.

*Details from Taylor & Francis Ltd.*

